# Electrical stimulation of the ventral tegmental area evokes sleep‐like state transitions under urethane anaesthesia in the rat medial prefrontal cortex via dopamine D_1_‐like receptors

**DOI:** 10.1111/ejn.14665

**Published:** 2020-02-24

**Authors:** Sabine Gretenkord, Bas M. J. Olthof, Myrto Stylianou, Adrian Rees, Sarah E. Gartside, Fiona E. N. LeBeau

**Affiliations:** ^1^ Biosciences Institute Medical School Newcastle University Newcastle Upon Tyne UK

**Keywords:** Oscillations, REM‐like, slow wave activity, Up state

## Abstract

The role of dopamine in regulating sleep‐state transitions during, both natural sleep and under anaesthesia, is still unclear. Recording in vivo in the rat mPFC under urethane anaesthesia, we observed predominantly slow wave activity (SWA) of <1 Hz in the local field potential interrupted by occasional spontaneous transitions to a low‐amplitude‐fast (LAF) pattern of activity. During periods of SWA, transitions to LAF activity could be rapidly and consistently evoked by electrical stimulation of the ventral tegmental area (VTA). Spontaneous LAF activity, and that evoked by stimulation of the VTA, consisted of fast oscillations similar to those seen in the rapid eye movement (REM)‐like sleep state. Spontaneous and VTA stimulation‐evoked LAF activity occurred simultaneously along the dorsoventral extent of all mPFC subregions. Evoked LAF activity depended on VTA stimulation current and could be elicited using either regular (25–50 Hz) or burst stimulation patterns and was reproducible upon repeated stimulation. Simultaneous extracellular single‐unit recordings showed that during SWA, presumed pyramidal cells fired phasically and almost exclusively on the Up state, while during both spontaneous and VTA‐evoked LAF activity, they fired tonically. The transition to LAF activity evoked by VTA stimulation depended on dopamine D_1_‐like receptor activation as it was almost completely blocked by systemic administration of the D_1_‐like receptor antagonist SCH23390. Overall, our data demonstrate that activation of dopamine D_1_‐like receptors in the mPFC is important for regulating sleep‐like state transitions.

## Graphical abstract

Under urethane anaesthesia, activity in the PFC spontaneously switches between slow wave activity and rapid eye movement (REM)‐like, low‐amplitude fast (LAF) oscillations. Electrical stimulation of the ventral tegmental area (VTA) evokes LAF in the PFC. Transition to LAF evoked by stimulation of the VTA is blocked by systemic SCH23390, a D_1/5_ antagonist. Dopamine, acting via D_1/5_ receptors, contributes to SWA/LAF transitions demonstrating a new role for dopamine in sleep‐like state transitions.

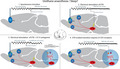

**Abbreviations**
AC: Anterior cingulate
Base: Baseline
CV: Coefficient of variation
DA: Dopamine
DP: Dorsopeduncular
DREADDS: Designer receptors exclusively activated by designer drugs
EEG: Electroencephalogram
GABA: Gamma‐aminobutyric acid
i.p.: Intraperitoneal
IL: Infralimbic
IQR: Interquartile range
ISI: Interspike interval
KS: Kolmogorov–Smirnov
LAF: Low‐amplitude‐fast (activity)
mPFC: Medial prefrontal cortex
Non‐REM: Non‐rapid eye movement (sleep)
PBS: Phosphate buffered saline
PrL: Prelimbic
REM: Rapid eye movement
s.e.m.: Standard error of the mean
*SD*: Standard deviation
SWA: Slow wave activity
UDS: Up–Down state
VTA: Ventral tegmental area

## 1 INTRODUCTION

Dopamine neurons in the VTA are important in controlling emotional and motivational behaviour (reviewed in Morales & Margolis, 2017; Schultz, 2015), but their role in regulating sleep and wakefulness remains controversial (Jones, 2020; Monti & Monti, 2007; Oishi & Lazarus, 2017) . Sleep consists of two distinct patterns of cortical activity, rapid eye movement (REM) sleep and non‐rapid eye movement (non‐REM) sleep, that can be readily distinguished in local field potential recordings within the neocortex or from surface electroencephalogram (EEG) electrodes.

Rapid eye movement sleep is distinguished by a low amplitude, faster network activity with oscillatory activity in the theta (4–8 Hz) and gamma (30–80 Hz) bands. In contrast, non‐REM sleep is characterised by large regular slow oscillations (~0.5–2 Hz) that are highly synchronous across the cortex, and sleep spindles at ~9–15 Hz (Isomura et al., 2006; Ruiz‐Mejias, Ciria‐Suarez, Mattia, & Sanchez‐Vives, 2011; Volgushev, Chauvette, Mukovski, & Timofeev, 2006). The cortical slow oscillations seen in non‐REM sleep are characterised by transitions between Up and Down states evident in the local field potential or EEG. During Up and Down states, the membrane potential of cortical pyramidal cells is depolarised and hyperpolarised, respectively, with firing occurring predominantly during the depolarised Up state (Bartho et al., 2014; Chauvette, Volgushev, & Timofeev, 2010; Steriade, Amzica, & Contreras, 1996; Steriade, Nunez, & Amzica, 1993). All mammals regularly alternate between non‐REM and REM states throughout the sleep period, albeit with different cyclical patterns in different species (Brown, Basheer, McKenna, Strecker, & McCarley, 2012; Hobson & Pace‐Schott, 2002; Pace‐Schott & Hobson, 2002).

Several studies have shown that the regular SWA observed under urethane anaesthesia displays many of the features characteristic of the non‐REM slow oscillations associated with deep sleep (Bartho et al., 2014; Beltramo et al., 2013; Chauvette et al., 2010; Destexhe, Contreras, & Steriade, 1999; Steriade et al., 1993). Although under anaesthesia SWA predominates, transitions to REM‐like activity have also been observed (Clement et al., 2008; Pagliardini Funk & Dickson, 2013a; Pagliardini Gosgnach & Dickson, 2013b; Pagliardini, Greer, Funk, & Dickson, 2012; Sakata & Harris, 2012; Zhurakovskaya et al., 2019).

What systems regulate sleep‐state transitions is still unclear, although there is evidence that several neurotransmitters including glutamate and GABA, as well as the cholinergic and noradrenergic systems all play a role in sleep and arousal (Hobson & Pace‐Schott, 2002; Jones, 2020; Monti et. al., 2013; Pace‐Schott & Hobson, 2002). Although early studies suggested that dopamine played no role in regulating sleep–wake behaviour, more recent evidence points to the contrary (for reviews see Jones, 2020; Monti & Monti, 2007; Oishi & Lazarus, 2017). Several recent studies have led to a renewed interest in the potential role of dopamine in modulating sleep–wake transitions (Luo et al., 2018; Oishi et al., 2017; Qu et al., 2010; Sun et al., 2017; Taylor et al., 2016). Electrical stimulation of the VTA has been shown to induce reanimation from general anaesthesia in rats (Solt et al., 2014; Taylor et al., 2016). Two recent electrophysiological studies using optogenetic and chemogenic approaches (in the absence of anaesthesia) have demonstrated a critical role for dopamine neurons in the VTA in regulating both the transition to the awake state and the maintenance of wakefulness (Eban‐Rothschild, Rothschild, Giardino, Jones, & Lecea, 2016; Oishi & Lazarus, 2017).

Neurons from the VTA heavily innervate the mPFC (Oades & Halliday, 1987; Swanson, 1982). The VTA is also active during REM sleep when dopamine neurons fire in bursts of spikes up to 40 Hz (Dahan et al., 2007). In rats under urethane anaesthesia, electrical stimulation of the VTA can lead to membrane depolarisation in presumed pyramidal cells in the mPFC (Lewis & O'Donnell, 2000). Onn and Wang (2005) also reported VTA stimulation‐evoked membrane depolarisation in mPFC neurons recorded under anaesthesia and showed that dopamine D_1_‐like receptors were involved in the depolarisation of pyramidal cells to the Up state. Thus, there is evidence that VTA stimulation can modulate pyramidal cell firing in the cortex, however, it remains unclear whether this regulates transitions between SWA and REM‐like sleep states.

In this study, we combined field and extracellular single‐unit recordings in the mPFC with electrical stimulation of the VTA to test the hypothesis that under anaesthesia projections from the VTA to the mPFC are involved in the switching between the slow oscillations of SWA to REM‐like, low‐amplitude‐fast oscillatory activity. In view of the different functional and anatomical differences between mPFC subregions (Heidbreder & Groenewegen, 2003; Kesner & Churchwell, 2011), we have recorded in all subregions: the anterior cingulate (AC), prelimbic (PrL), infralimbic (IL) and dorsopeduncular (DP) cortices. We found that electrical stimulation of VTA consistently abolished SWA and induced a low‐amplitude‐fast (LAF) network rhythm, similar to the REM‐like sleep state, that was evident along the dorsoventral extent of the mPFC. Systemic injection of a D_1_‐like dopamine receptor antagonist blocked the effects of VTA stimulation in all mPFC subregions. Our data demonstrate that dopamine, via D_1_‐like receptors, mediates the VTA stimulation‐induced transitions from SWA to a REM‐like state.

## 2 METHODS

### 2.1 Animals

All procedures described were performed in accordance with the UK Animals (Scientific Procedures) Act 1986 and the European Union Directive 2010/63/EU. Male Hooded Lister rats (Charles River Laboratories) were housed at Newcastle University's animal facility in a temperature‐ and humidity‐controlled environment consistent with the ARRIVE (Animal Research: Reporting of In Vivo Experiments) guidelines. Rats were kept in an enriched environment (cage toys) under a 12‐hr light–dark cycle (lights on 7 a.m. – 7 p.m.) with access to food and water ad libitum. Rats were housed up to four per cage and were allowed a week of acclimatisation before the experiment. Experiments were commenced ~2 hr into the light (sleep) phase of the circadian cycle.

### 2.2 Anaesthesia and surgery

Rats weighing 250–330 g were anaesthetised with urethane (Sigma‐Aldrich). An initial dose of 1.5–1.9 g/kg was administered by intraperitoneal (i.p.) injection. Additional doses of 0.5 g/kg i.p. were given every half hour until a surgical plane of anaesthesia (confirmed by absence of the pedal withdrawal reflex) was achieved. The animal was fixed in a stereotaxic frame (Kopf). A heating pad with feedback temperature control via a rectal probe (Harvard Apparatus) maintained the core temperature of the rat at 36.8°C. A pulse oximeter (Physiosuite, Kent Scientific) was attached to the animal's hind paw to measure blood oxygen saturation. The animal breathed spontaneously, but to maintain an oxygen saturation of >90%, medical oxygen (BOC Industrial Gases, UK) was supplied through a tube mounted on the incisor bar of the stereotaxic frame. A skin incision was made in the scalp and infused with lidocaine before the periosteum was retracted to expose the skull. Craniotomies were drilled above the mPFC of both hemispheres (co‐ordinates from bregma AP + 2.3–2.5 mm, ML +0.5 mm) and above the VTA (AP −5.8 mm, ML + 0.6 mm, left hemisphere). A concentric bipolar stimulating electrode (NE‐100 concentric, 50 mm shaft length, outer diameter 500 μM, contact separation 1 mm; Rhodes Medical Instruments) was implanted into the VTA (DV – 8.0 mm), and recording electrodes were implanted in the mPFC as described below.

### 2.3 Data recording and acquisition

Two different recording configurations were used in this study. In some experiments, recordings were made from the right prelimbic region of the mPFC using glass‐coated tungsten electrodes implanted using a remote‐controlled stepper microdrive. Tungsten electrodes were connected to a headstage, and the signal was amplified (×1,000) and filtered (0.1–10 kHz) using a preamplifier (DAM‐80, World Precision Instruments). The signal was further low‐pass filtered with a cut‐off frequency of 500 Hz (TDT system 2, Tucker Davis Technologies) to extract the local field potential and digitised by a Micro‐1401 (Cambridge Electronic Design) at a sampling rate of 2,000 Hz. In other experiments, multi‐channel recordings were made in all subregions of the mPFC in both hemispheres simultaneously with dual shank (1 mm separation) 16‐channel silicon probes (8 recording sites per shank, 500 µm inter‐site spacing; E16‐500–S02–1000‐L7.5, Atlas Neuroengineering). Silicon probes were lowered (with one shank in each hemisphere) to a depth of 5.3 mm using a one‐axis oil‐filled hydraulic micromanipulator (Narishige). Before insertion, the probes were coated with a fluorescent dye (DiI)(1,1’‐dioctadecyl‐3,3,3’,3’‐tetramethylindocarbocyanine; Molecular Probes, Eugene, Oregon, USA), dissolved in DMSO (1.5–2.5 mg/ml)) to mark the electrode tracks. For local field potential recordings, two recording contacts were located in each subregion of the mPFC: AC, PrL, IL and DP in both hemispheres as previously described (Gretenkord, Rees, Whittington, Gartside, & LeBeau, 2017). Local field potential recordings were similar in both hemispheres, so all statistics and data presented are only for recordings from the left hemisphere (ipsilateral to the VTA stimulation). For each channel, the signal was passed through a unity‐gain headstage (Plexon) and then amplified (×1,000) and filtered (0.07–300 Hz for field potential; 0.15–9 kHz for spikes) by a Plexon preamplifier (Plexon). The continuous local field potential was digitised at 1,000 Hz and recorded on a PC (Dell) running Plexon software (Sort Client).

### 2.4 Electrical stimulation of the VTA

Stimulation patterns were programmed on a Master‐8 stimulator (A.M.P.I, Jerusalem, Israel) and delivered via an Iso‐Flex stimulus isolator (A.M.P.I, Jerusalem, Israel). VTA electrical stimulation consisted predominantly of a 50 Hz/30 s stimulation protocol with a continuous train of 1,500 biphasic pulses (0.1 ms duration, 20 ms interval). To examine the current intensity response relationship and establish stimulation parameters to be used in drug experiments, the current was slowly increased from ~ 0.1 mA in 0.02 to 0.05 mA steps, until a clear, but submaximal response to the stimulation was observed, with a similar latency to onset of LAF (See below). Electrical stimulation of the VTA was then applied at 10 min intervals. In some experiments, a burst stimulation pattern was used (30 s duration, 5 biphasic pulses per burst, intra‐burst frequency 25 Hz, 1 s inter‐burst‐interval).

### 2.5 Drug administration

To investigate the role of dopamine D_1_‐like receptors, the response to VTA electrical stimulation was assessed during three baseline (Base1‐3) stimulations and at three time points (10, 20 and 30 min) after systemic (i.p.) administration of either saline (Sal1‐3), or the dopamine D_1_‐like antagonist SCH23390 (SCH1‐3). Two doses of SCH23390 were assessed (“low dose”: 0.3 mg/kg, or “high dose”: 0.6 mg/kg).

### 2.6 Histological verification of recording site position

After the experiment, the rat was killed by injection with Euthatal (200 mg/ml i.p.). The brain was removed from the skull and postfixed in 4% paraformaldehyde (PFA) in 0.1M phosphate buffer saline (PBS) at 4°C for a minimum of 12 hr and cryoprotected in 30% sucrose solution. Coronal sections (60–100 μm) were cut on a cooled vibratome (Zeiss Hyrax V50, Zeiss, Oberkochen, Germany) and collected in 0.1M PBS. Cresyl violet staining (see below) was used to verify the position of single channel tungsten electrodes, and green fluorescent Nissl stain (NeuroTrace 500/525, Molecular Probes, Eugene, Oregon, USA) or bisbenzimide H33258 (Sigma‐Aldrich, St. Louis, MO, USA) was used for the silicone probes marked with DiI. Following staining, sections were mounted and coverslipped using Vectashield HardSet mounting medium (Vector Labs LTD., Peterborough, UK).

### 2.7 Verification of stimulation sites

For verification of the stimulation electrode placement in the VTA, either a cresyl violet staining protocol or a tyrosine hydroxylase (TH) immunohistochemistry protocol was used. For TH immunohistochemistry, free floating sections were washed in PBS, incubated in 0.3% H_2_O_2_ for 30 min and permeabilised with 1% Triton‐X (Sigma‐Aldrich) for 20 min before being incubated overnight at 8°C with mouse anti‐TH‐16 (Sigma‐Aldrich Cat. number T2928RRID:AB 477,569) (1:10,000, in diluent, 3% bovine serum albumin, 1.8% lysine in PBS). The next day, sections were washed and incubated for 2 hr at room temperature in biotinylated conjugated horse anti‐mouse IgG (Vector laboratories) (1:100 in diluent), followed by horse radish peroxidase (HRP) conjugated streptavidin (1:300 in PBS) or HRP avidin D (1:100 in PBS) at room temperature. Finally, sections were incubated for 5–10 min in diaminobenzidine (Sigma‐Aldrich). Sections were mounted on gelatin‐subbed slides and allowed to dry before being dehydrated in ethanol, cleared in Histoclear (National diagnostics) and coverslipped with Entellan (Sigma‐Aldrich). For cresyl violet staining, sections were mounted on gelatin‐subbed slides and allowed to dry before being dipped in cresyl violet, washed in water, dehydrated in ethanol, cleared in Histoclear and coverslipped with Entellan.

### 2.8 Data analysis

#### 2.8.1 Up–down state detection

All data analysis was performed offline using custom MATLAB (Mathworks) scripts. Up–Down state detection was performed using the phase of the slow oscillation, as described previously (Massi et al., 2012), except that the Hilbert transform (rather than the wavelet transform) was used to calculate the phase of the slow oscillation (as described in Gretenkord et al., 2017). The local field potential was first bandpass filtered (0.1–0.9 Hz), and the instantaneous phase ϕ(t) was calculated using the Hilbert transform. The threshold to discriminate between Up and Down states was $cos(\phi \left(t\right)=0)$. To qualify as an Up state, the average amplitude over all channels was required to be larger than 0.5 mV for a duration > 300 ms.

#### 2.8.2 Detection of LAF activity

**Figure 1:**
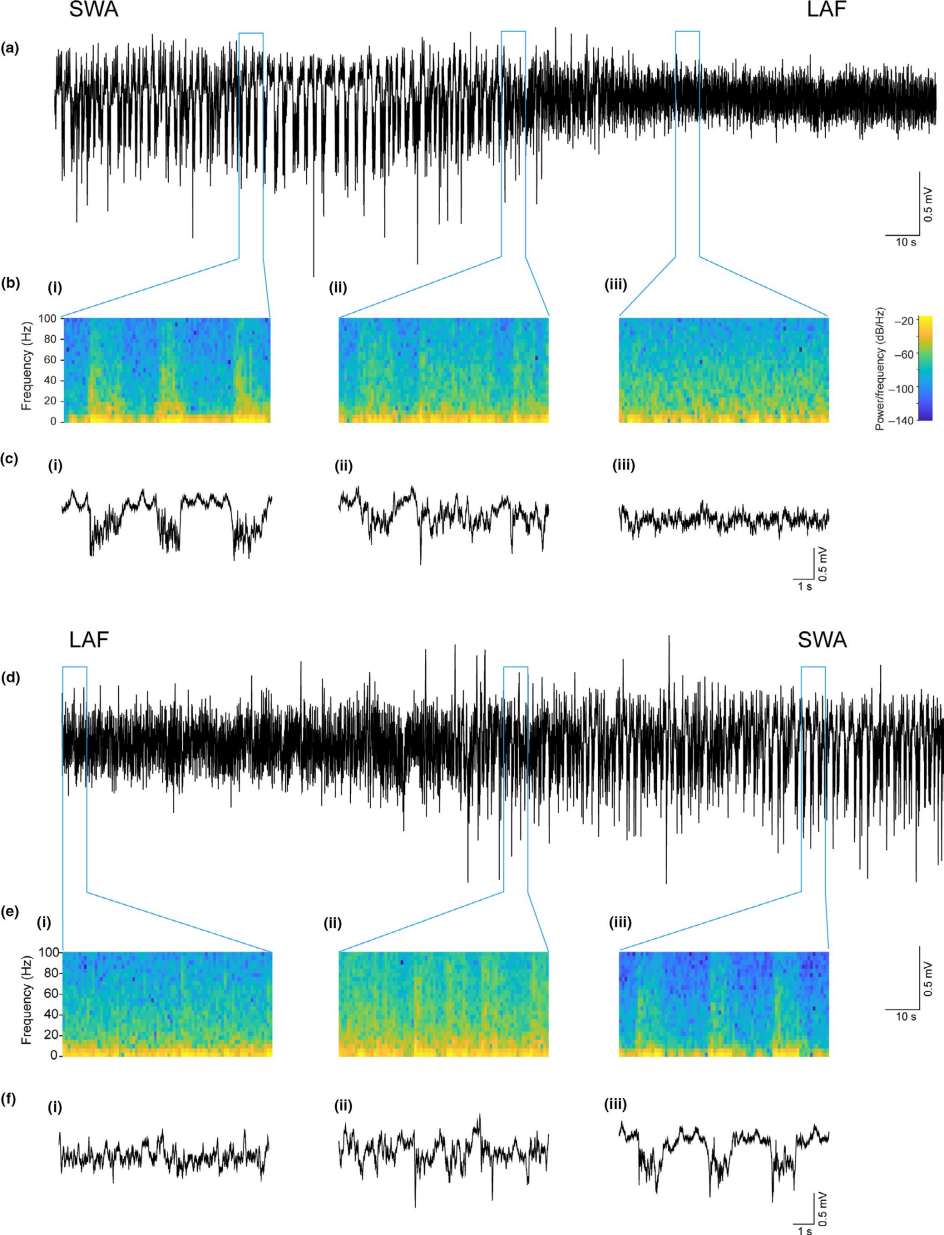
Spontaneous transitions from SWA to LAF activity under urethane anaesthesia. (a) Local field potential trace recorded in the PrL subregion of the mPFC shows oscillatory activity changes from SWA activity to LAF. (b) Spectrograms and (c) expanded traces taken as indicated from the local field potential in (a) during (i) SWA (ii), during the transitions from SWA‐LAF activity and (iii) during LAF activity. (d) Local field potential trace recorded in the PrL subregion of the mPFC shows oscillatory activity changes from LAF activity to SWA. (e) Spectrograms and (f) expanded traces taken as indicated from the field potential in (d) during (i) LAF (ii), during the transitions from LAF activity to SWA and (iii) during SWA [Colour figure can be viewed at wileyonlinelibrary.com]

Local field potential segments were aligned to the stimulation period, and the LAF activity induced by VTA stimulation was detected from the amplitude of the slow oscillation. Local field potential segments were filtered using a 0.1–2 Hz 2nd order Butterworth bandpass filter. The analytical signal of the filtered local field potential was calculated using the Hilbert transform, and an amplitude envelope was calculated as the complex modulus (magnitude) of the analytical signal. The amplitude envelope was smoothed using a moving‐average filter with a 3‐s window. Visual inspection of the analysis confirmed that a 3‐s smoothing window prevented most small, brief fluctuations in signal amplitude (including Up states during the SWA) from being detected as LAF activity. The mean amplitude in a one‐minute period immediately before stimulation onset (baseline) was calculated, and LAF activity was defined as activity with an amplitude <50% of the mean baseline amplitude. To capture both the latency to onset of the LAF activity transition, and interruptions in the evoked LAF activity, our main outcome measure was *time in LAF activity = *the total time spent in LAF activity during the stimulation period. In the case of spontaneous transitions, which were slower in onset than the VTA stimulation‐evoked transitions, the mean baseline amplitude was calculated for a period of clear SWA several minutes before the emergence of LAF activity (Figure 1).

#### 2.8.3 Single‐unit analysis

Single units recorded in the mPFC were sorted using principal component analysis in Offline Sorter (Plexon). Only units that could be well distinguished were included in the analysis. In most cases, one unit per channel was recorded although occasionally two or even three units could be isolated. For all isolated units, the percentage of spikes on the Up state was calculated in a 5‐min baseline period of SWA, either a few mins before a spontaneous transition to LAF activity, or immediately before VTA stimulation. The distribution of interspike intervals (ISIs) in SWA and LAF activity was calculated for a 25‐min period (bin width 50 ms) encompassing a spontaneous transition from SWA to LAF activity, or a 15 min period encompassing the third baseline (Base3) VTA stimulation. Intervals were divided into those occurring during SWA and those during LAF activity (detected as described above). The coefficient of variation (CV) of the interspike intervals (ISIs) was calculated for units recorded during periods of SWA and LAF. ISIs that were not contained within a single Up state (longer than 1,500 ms) were excluded so that the calculated CV refers only to firing on the Up state of SWA. During both SWA and LAF activity, the mean and standard deviation (*SD*) of the ISI for individual units were calculated, and the CV for each unit was calculated as σ ISI/µ ISI. The median and interquartile ranges of the CVs for all units are presented.

#### 2.8.4 Statistics

Non‐parametric statistical methods in SPSS were used for all analysis, and data were presented as median and interquartile range (IQR). Between groups, comparisons were made using Kruskal–Wallis test for independent samples. Within group comparisons were made using Friedman's one‐way ANOVA for related samples, and significant ANOVA findings were followed up with Wilcoxon signed rank test for related samples. Differences between distributions were tested using the Kolmogorov–Smirnov (KS) test. Statistical significance was indicated with exact P‐values in the text.

## 3 RESULTS

### 3.1 Spontaneous state transitions occur under urethane anaesthesia

Prior to investigating the impact of electrical VTA stimulation on activity in the prefrontal cortex, we recorded the local field potential and spiking activity of neurons in all subregions of the mPFC in urethane‐anaesthetised rats. With a deep surgical plane of anaesthesia, SWA was the predominant activity pattern observed, but occasional spontaneous transitions back and forth between SWA and a low‐amplitude‐fast (LAF) activity pattern were observed (Figure 1a‐f), similar to those reported previously (Clement et al., 2008; Sakata & Harris, 2012). However, these spontaneous transitions were, as others have reported, very rare (Fenik, Marchenko, Davies, & Kubin, 2012; Rukhadze, Fenik, Branconi, & Kubin, 2008) , with 1–2 events occurring in only 6/18 rats used in this study. During SWA, fast oscillatory activity, mainly in the beta (15–30 Hz) and gamma (30–80 Hz) bands (Figure 1bi and eiii), was associated with the Up state (downward deflection in the extracellular LFP), while during the LAF activity, fast oscillations were more continuous (Figure 1biii and ei).

**Figure 2:**
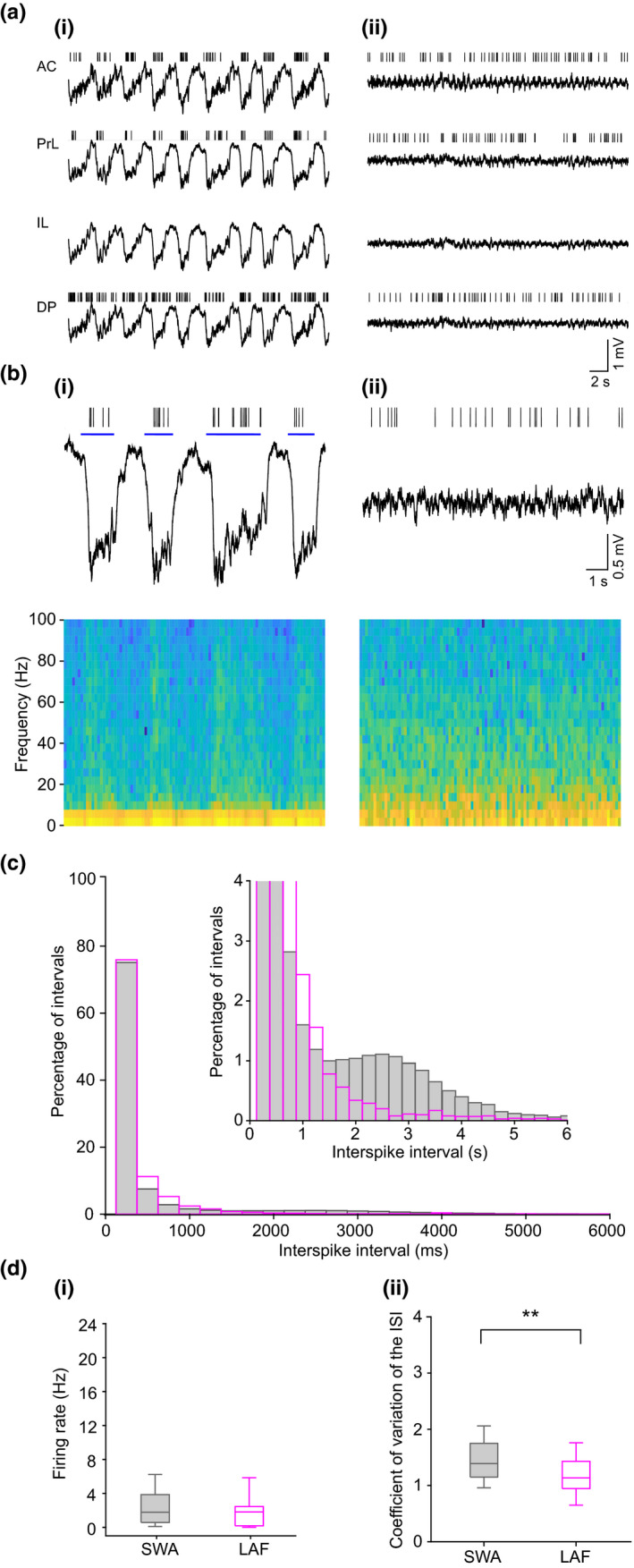
Spontaneous transitions from SWA to LAF activity are associated with changes in mPFC unit firing properties. (ai) SWA activity recorded simultaneously in all subregions of the mPFC including the anterior cingulate (AC), prelimbic (PrL), infralimbic (IL) and dorsopeduncular (DP) cortices. Vertical lines show individual spike patterns from single neurons recorded simultaneously in mPFC subregions (except IL). Firing during SWA is phasic with spikes restricted to the Up state (corresponding to the downward deflection of the local field potential). (aii) Local field potential during the same recording session showing spontaneous LAF activity was present simultaneously in all mPFC subregions. The same neurons shown in (ai) now fire tonically during spontaneous LAF activity. (b) Expanded time scale showing the PrL channel recording during (bi) SWA and (bii) spontaneous LAF activity with corresponding spectrograms. During SWA, spiking is restricted to the Up state (blue line), but continuous firing occurs during spontaneous LAF activity. (c) Histogram of ISI distribution, 19 units (grouped in 250 ms bins) for SWA (grey) and LAF activity (magenta). Inset (truncated for clarity) shows more frequent long ISIs in SWA reflecting the phasic firing with spikes restricted to the Up state. di) Box plot shows the median (IQR) firing rate (Hz) for all units (*n* = 19) recorded during SWA and spontaneous LAF activity were not significantly different. (dii) Box plot of CV for units (*n* = 16) shows a significant (*p* = .003) decrease in CV during spontaneous LAF activity (3 units which stopped firing during LAF were removed) [Colour figure can be viewed at wileyonlinelibrary.com]

The spontaneous sleep‐state transitions occurred simultaneously in all subregions throughout the dorsoventral extent of the mPFC and were associated with a significant change in single neuron spiking activity in the mPFC (Figure 2a,b). During SWA, all mPFC neurons recorded exhibited phasic firing with spikes occurring almost exclusively on the Up state (mean 89.4% ± 1.32 spikes on Up state, *n* = 19, Figure 2ai–bi). In contrast, during spontaneous periods of LAF activity, neurons switched to a tonic pattern of spiking (Figure 2aii–bii). The interspike interval (ISI) histogram (Figure 2c) showed that during SWA, there are both short ISIs (reflecting spikes on the Up state) and long ISIs (>1,500 ms, reflecting intervals between spikes at the end of one Up state and the beginning of the next). In contrast, during LAF activity, in the absence of such state changes, fewer long ISIs occurred (Figure 2c). Overall, the distribution of ISIs (grouped in 50 ms bins) during the spontaneous LAF activity was significantly different from that during SWA (*p* = 1.968 x 10^–7^, KS test). The median firing frequency (Figure 2di) of all units in SWA was 1.78 (IQR 0.54–4.02) Hz (*n* = 19), which was not significantly different to that during spontaneous LAF activity, 1.81 (IQR 0.17–2.57) Hz (*p* = .376, Wilcoxon signed rank test). To calculate the regularity of firing using the coefficient of variation (CV) of the ISIs, we excluded the long intervals (>1,500 ms) reflecting intervals between spikes in different Up states, thus comparing firing only on the Up state with firing during LAF activity. Our results showed that the median CV (Figure 2dii) was significantly higher during SWA than during LAF activity, 1.39 (IQR 1.14–1.78) versus 1.13 (IQR 0.94–1.49), *n* = 16 ((3 units that stopped firing during LAF were excluded), *p* = .003, Wilcoxon signed rank test)). The lower CV in LAF activity, which occurs despite the removal of long intervals, indicates that unit firing was more regular during LAF activity.

### 3.2 VTA stimulation induces a transition from SWA to LAF activity

**Figure 3:**
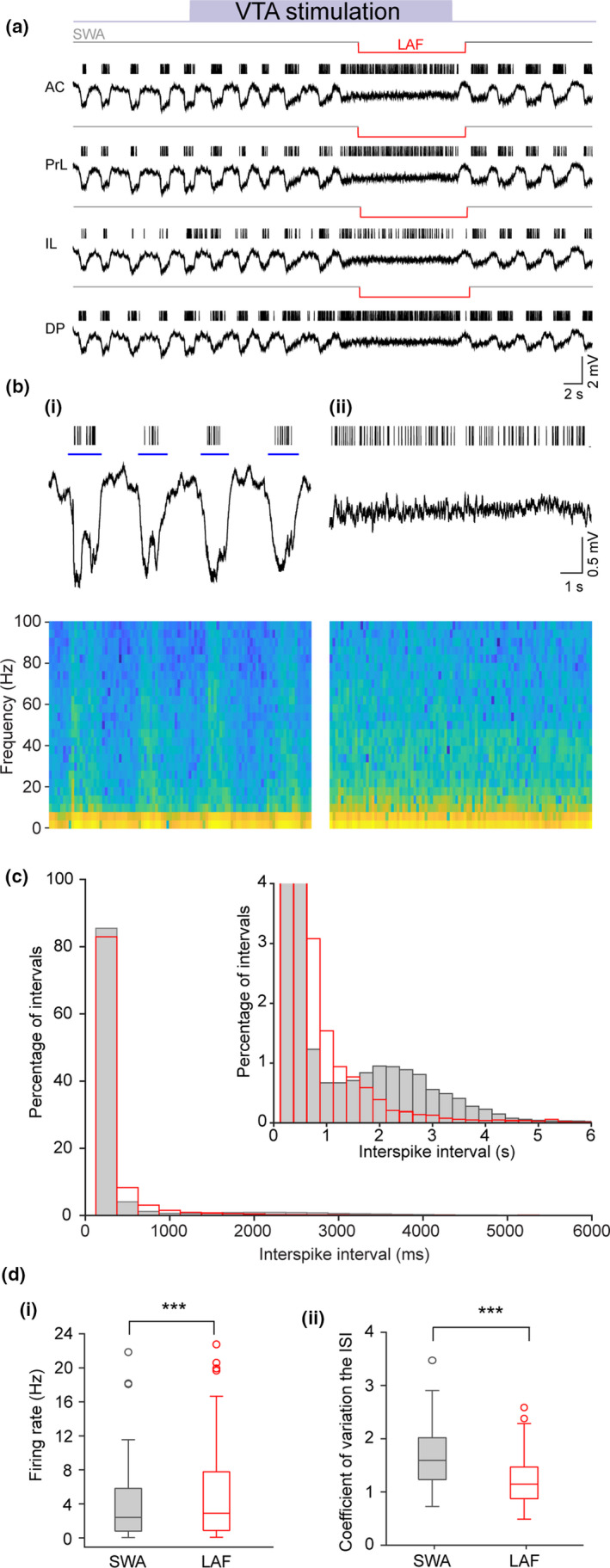
Electrical stimulation of the VTA evokes a state switch from SWA to LAF activity. (a) Local field potential recordings for each mPFC subregion; anterior cingulate (AC), prelimbic (PrL), infralimbic (IL) and dorsopeduncular (DP) cortices. VTA electrical stimulation (purple bar) evoked a transition to LAF activity simultaneously in all mPFC subregions (LAF detection indicated by text above red line).Vertical lines show unit firing for one neuron from each mPFC subregion and illustrates the transition from phasic firing on the Up state to tonic firing during LAF activity. (b) Expanded time scale showing the PrL channel recording during (bi) SWA and (bii) VTA stimulation‐evoked LAF activity with corresponding spectrograms. During SWA activity spiking is restricted to the Up state (blue line) but continuous firing occurs during VTA stimulation‐evoked LAF activity. (c) Histogram of ISI distribution (grouped in 250 ms bins) for SWA (grey) and VTA stimulation‐evoked LAF activity (red). Inset (truncated for clarity) shows more frequent long ISIs in SWA reflecting the phasic firing with spikes restricted to the Up state. (di) Box plot shows the median (IQR) for all units (*n* = 62) showing firing rate significantly increased (*p* = .000098) during VTA stimulation‐evoked LAF activity. (dii) Box plot shows the median (IQR) for all units showing a significant decrease in CV during VTA stimulation‐evoked LAF activity (*p* = 1.44 x 10^−10^, *n* = 56, (6 units that either stopped firing during LAF or had only ISI's > 1,500 ms were removed)) [Colour figure can be viewed at wileyonlinelibrary.com]

To investigate the potential role of the VTA in mediating sleep‐state transitions, we combined electrical stimulation of the VTA with extracellular recordings in the mPFC. To prevent spontaneous transitions to LAF activity, we used a supplementary dose of urethane (0.3 mg/kg) as we have previously found that this produced an extended period (~2–3 hr) of continuous SWA without spontaneous transitions (Gretenkord et al., 2017). Electrical stimulation of the VTA (regular 50 Hz, 30 s, 0.1–0.6 mA) resulted in clear transitions from SWA to LAF activity (Figure 3). The transitions to LAF activity occurred either immediately following the start of the VTA stimulation, or after a short delay (e.g. ~15 s in Figure 3a). The LAF activity then persisted for the remainder of the stimulation period with SWA re‐emerging either rapidly on cessation of stimulation as in the example shown (Figure 3a), or following a few seconds delay. As seen with the spontaneous transitions outlined above (Figure 2), the VTA stimulation‐evoked switch from SWA to LAF activity evoked by VTA stimulation occurred in all subregions of the mPFC simultaneously and was associated with a transition from phasic to tonic firing (Figure 3a‐b).

We recorded a total of 64 single units across the mPFC subregions during SWA in nine animals. During SWA activity, the vast majority of these units (62/64) fired phasically with spikes almost exclusively on the Up state. The two units which fired mostly on the Down state were excluded from the subsequent analysis for clarity. In the 62 remaining units, the average percentage firing restricted only to the Up state was 95.1 ± 1.0% with very little firing in the Down state. There were no significant differences in firing rate across the mPFC subregions during SWA (Table 1, *n* = 62); therefore, all mPFC units were combined for further analysis. The ISI histogram (Figure 3c) again showed that during SWA, there were both short and long ISIs, with the latter again reflecting the time between one Up state and the next. However, during LAF activity evoked by VTA stimulation, there was a reduction in the proportion of long intervals and, as was the case for spontaneous transitions reported above (Figure 2c), the distributions of intervals (grouped in 50 ms bins) were significantly different (*p* = .000074, KS test).

**Table 1:** Firing rates (Hz) in different mPFC subregions during SWA

	AC	PrL	IL	DP
*N*	14	11	20	17
Median	1.26	3.33	2.0	5.70
IQR	0.6–5.7	1.0–5.3	1.1–4.0	0.5–10.7

Note

AC, anterior cingulate cortex; DP, dorsopeduncular cortex; IL, infralimbic cortex; PrL, prelimbic cortex.

The changes seen in firing during LAF activity evoked by VTA stimulation were qualitatively similar to those observed during the spontaneous transitions to LAF activity, where firing also changed from a phasic to tonic pattern of activity (compare Figure 2b and Figure 3b). Firing rate was slightly, but significantly, higher during LAF activity evoked by VTA stimulation compared with SWA (Figure 3di) with a median for SWA of 2.41 (IQR 0.78 – 5.85) Hz versus 2.90 (IQR 0.82 – 7.81) Hz for LAF activity (*n* = 62, *p* = .000098, Wilcoxon signed rank test). As for spontaneous transitions to LAF activity (Figure 2), regularity of firing was also increased during stimulation‐evoked LAF activity, as indicated by a significant decrease in CV (Figure 3dii). Thus, the median CV in SWA was 1.59 (IQR 1.22 – 2.03) compared with 1.15 (IQR 0.87 – 1.48) for LAF activity ((*n* = 56 (6 units that stopped firing or had only intervals >1,500 ms after the switch to LAF activity were excluded), *p* = 1.44 x10^‐10^, Wilcoxon signed rank test)).

### 3.3 VTA stimulation‐evoked LAF activity is stimulus dependent

**Figure 4:**
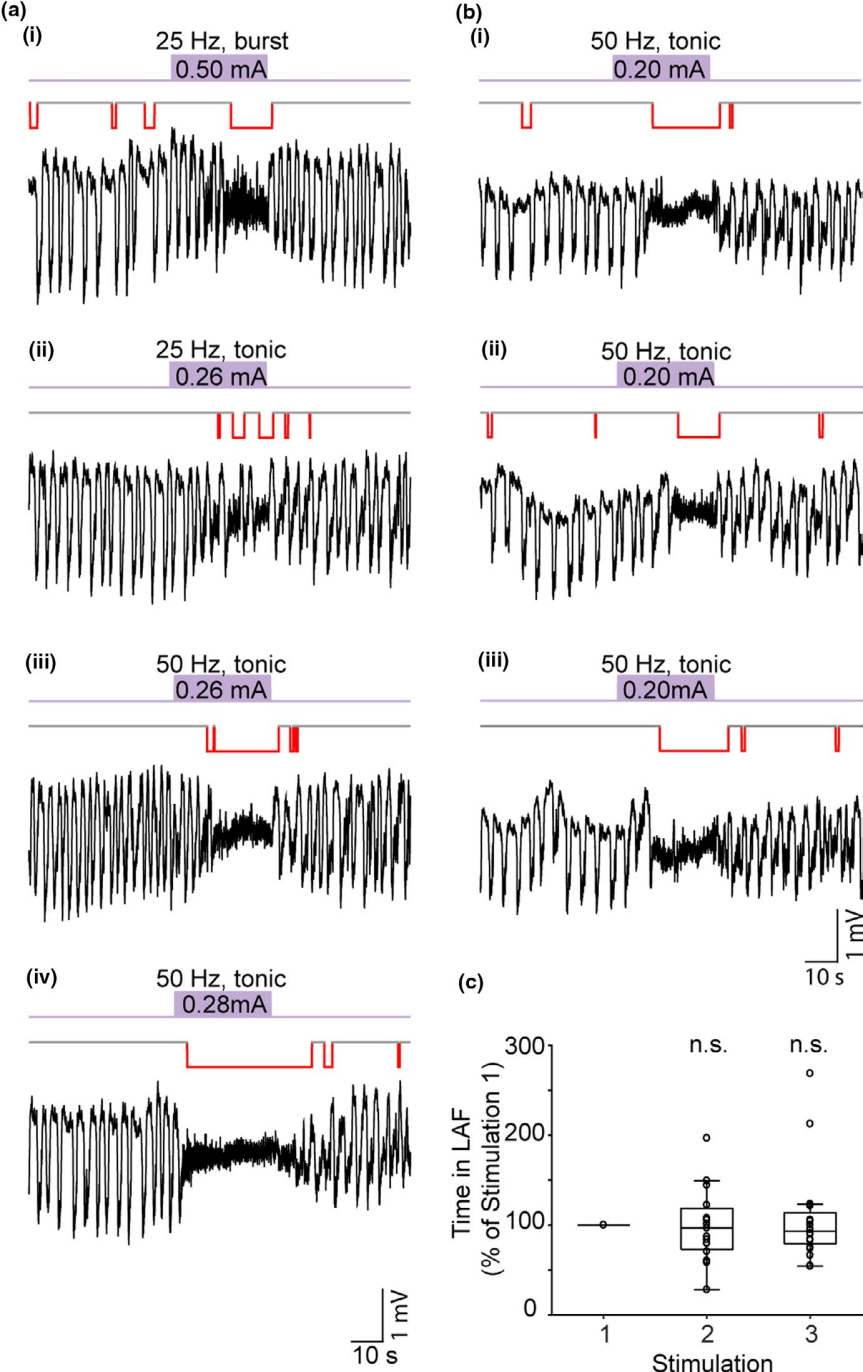
Low‐amplitude‐fast (LAF) activity can be induced by different VTA stimulation parameters (LAF activity indicated by red lines). (a) Extracellular field recordings from the PrL region showing that during SWA VTA stimulation (purple bar) at 25 Hz with either (ai) a burst pattern or (aii) regular stimulation pattern could evoke short periods of LAF activity. Increasing the VTA stimulation frequency to 50 Hz (aiii) elicited a longer period of LAF activity, after a short delay, which returned to SWA on cessation of the stimulation. Increasing the stimulus intensity to 0.28 mA (aiv) then caused a rapid transition to LAF that lasted a few seconds after cessation of the stimulation. (bi–iii) Three repeated 30 s VTA stimulations (50 Hz regular, 0.2 mA) at 10 min intervals evoked similar transitions from SWA to LAF activity. (c) Box plot shows no significant difference in median (IQR) time in LAF activity for the group data (*n* = 19 animals) for 3 repeated VTA stimulations at the same intensity [Colour figure can be viewed at wileyonlinelibrary.com]

The transition from SWA to LAF activity evoked by regular VTA stimulation at 50 Hz occurred either immediately after stimulation or with a short delay of ~10–15 s after stimulus onset (Figures 3 and 4). Transitions from SWA to LAF activity could also be evoked by a burst pattern of stimulation (Figure 4ai), and by lower frequency (25 Hz) regular VTA stimulation (Figure 4aii). Furthermore, with increasing stimulus intensity, the latency to the onset of LAF activity was reduced (compare Figure 4aiii vs. 4Aiv), and in some cases, the transition to LAF occurred immediately.

Before assessing the effects of dopamine modulation, we first established that the transitions to LAF activity evoked by VTA stimulation were reproducible (Figure 4b‐c). We specifically selected a stimulus intensity that evoked LAF activity with a short delay to enable us to observe either increases or decreases in the duration of LAF activity after dopamine antagonist administration. Three stimulations of the VTA (with the same current and intensity) at 10‐min intervals evoked periods of LAF activity of similar duration (Figure 4b). There was no significant difference in the time spent in LAF activity between the three stimulations (median stimulus 1 = 19.8 (IQR 16.4–23.3) s; stimulus 2 = 17.7 (IQR 14.4–19.3) s; stimulus 3 = 18.4 (IQR 14.6–21.3) s (*n* = 19)).

### 3.4 Dopamine D_1_‐like receptor blockade abolished the VTA‐evoked induction of LAF activity

**Figure 5:**
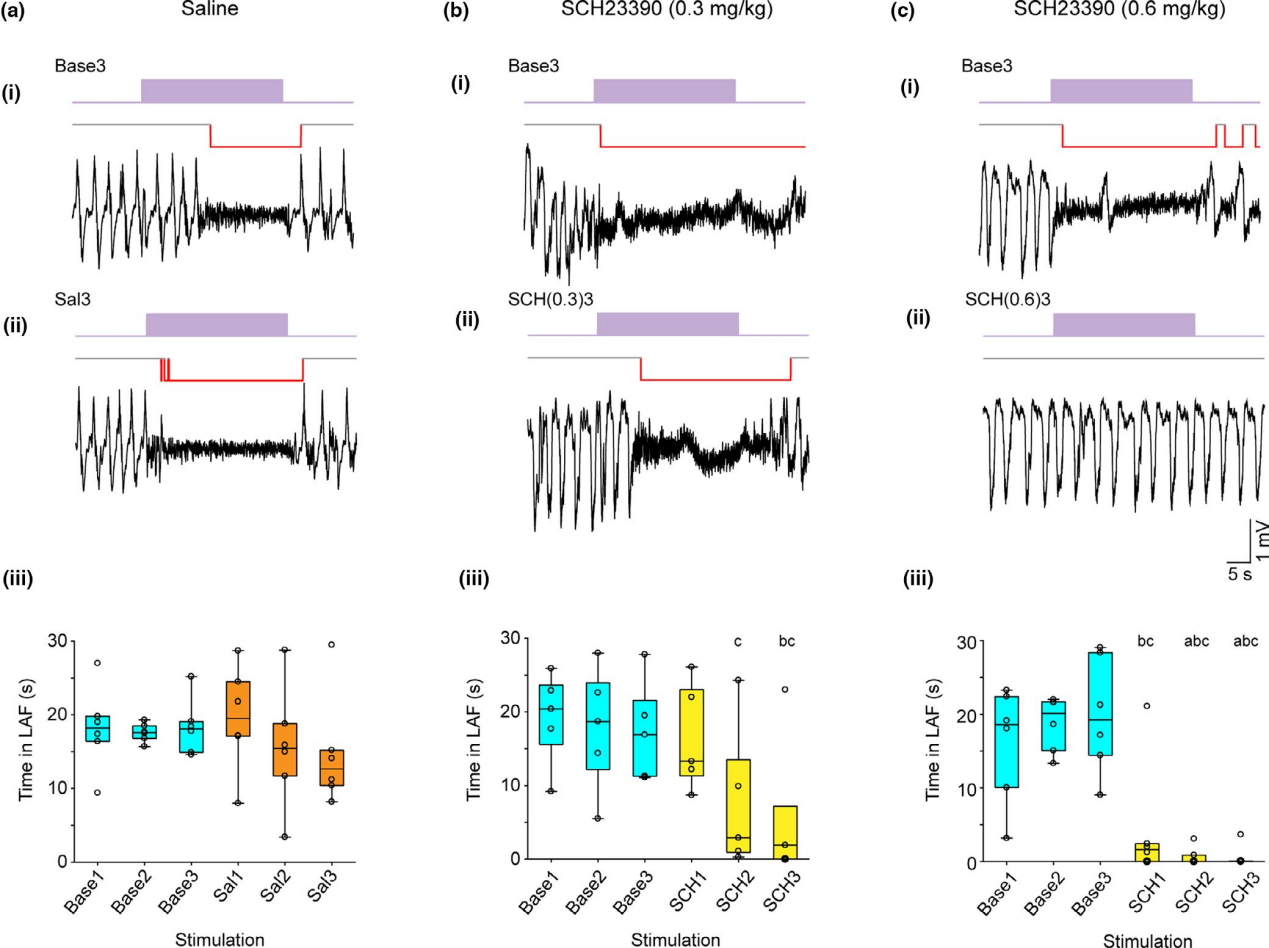
VTA stimulation‐evoked transition to LAF activityis dependent on D1‐like receptor activity. (a–c) Example local field potential recordings in the PrL cortex during SWA showing effects of VTA stimulation (50 Hz, 30 s, purple bar). (ai) Response to the third (Base3) of three baseline VTA stimulations given at 10 min intervals and (aii) the third VTA stimulation given at 10 min intervals following an i.p. injection of saline (Sal3). (aiii) Box plot shows the group data for the time in LAF activity (s) following all three baseline (Base1‐3) VTA stimulations and all three stimulations following an i.p. injection of saline (Sal1‐3). Systemic application of saline had no significant effects on the time in LAF activity. (bi) In a separate experiment response to the third baseline (Base3) of three baseline VTA stimulations given at 10 min intervals and (bii) the third VTA stimulation given at 10 min intervals following an i.p. injection of the D_1_‐like receptor antagonist SCH23390 at 0.3 mg/kg (SCH(0.3)3). (biii) Box plot shows the group data for the time in LAF activity (s) following all three baseline (Base1‐3) VTA stimulations and all three stimulations following an i.p. injection of SCH23390 (SCH1‐3). Systemic application of the D_1_‐like receptor antagonist SCH23390 at 0.3 mg/kg caused a significant reduction in the time in LAF activity 20 and 30 min post‐injection (SCH2 and SCH3). (ci) In a separate experiment, response to the third baseline (Base3) of three baseline VTA stimulations given at 10 min intervals and (cii) the third VTA stimulation given at 10 min intervals following an i.p. injection of SCH23390 at 0.6 mg/kg (SCH(0.6)3). (ciii) Box plot shows the group data for the time in LAF activity (s) following all three baseline (Base1‐3) VTA stimulations and all three stimulations following an i.p. injection of SCH23390 (SCH1‐3). Systemic application of the D_1_‐like receptor antagonist SCH23390 at 0.6 mg/kg caused a rapid and significant reduction in the time in LAF activity for all post‐injection stimulations (SCH1‐3). Key: c = different to Base3, b = different to Base2, a = different to Base1. For full statistical analysis see text [Colour figure can be viewed at wileyonlinelibrary.com]

Dopamine D_1_‐like receptors have been implicated in sleep‐state changes (Isaac & Berridge, 2003; Luo et al., 2018; Taylor et al., 2016). We tested their potential role in the VTA‐evoked transition to LAF activity by stimulating the VTA before and after systemic administration of the D_1_‐like receptor antagonist SCH23390 or saline (Figure 5). In animals injected with saline, there was no significant difference in the time spent in LAF activity evoked by three baseline stimulations and the three postsaline stimulations (Friedman's ANOVA, Figure 5ai–iii). However, in animals injected with a low dose of SCH23390, the time in LAF activity evoked by VTA stimulations after the drug administration was significantly reduced, compared with that evoked by stimulations before drug administration (Figure 5bi–iii). Thus, Friedman's ANOVA showed a significant effect of stimulation (*p* = .048), and post hoc analysis (Wilcoxon signed ranks test) showed significant differences between the pre‐drug stimulations and post‐drug stimulations: Base3 versus SCH(0.3)2, (*p* = .043) and Base2 and Base3 versus SCH(0.3)3, (*p* = .043). The higher dose of SCH23390 (0.6 mg/kg) had an even more dramatic effect causing a rapid and almost complete blockade of the effect of VTA stimulation (Figure 5ci‐iii). A Friedman ANOVA showed a significant effect of stimulation (*p* = .001), and a Wilcoxon signed rank test showed significant differences between the pre‐drug stimulations and all post‐drug stimulations: Base2 versus SCH(0.6)1,(*p* = .046) and Base3 versus SCH(0.6)1, (*p* = .028) and Base1‐3 versus SCH(0.6)2 and SCH(0.6)3, (*p* = .028 all comparisons). In order to account for possible changes that might occur due to repeated stimulations, or the passage of time, we also made comparisons between the Sal and SCH(0.3) groups and the Sal and SCH(0.6) group for all baseline and post‐injection time points. These analyses showed that there were no differences between groups in the baseline time points. While the differences between Sal and SCH(0.3) groups post‐injection failed to reach significance, the differences between Sal and SCH(0.6) groups were highly significant at all three post‐injection points (*p* = .016, *p* = .003 and *p* = .003 at post‐injection time points 1, 2 and 3, respectively). Overall, therefore, these results demonstrate that dopamine D_1_‐like receptors are involved in mediating the VTA stimulation‐evoked transition to LAF activity.

## 4 DISCUSSION

In this study, we showed that in rats under urethane anaesthesia, electrical stimulation of the VTA is sufficient to evoke a transition in the mPFC local field potential from non‐REM‐like slow oscillations of SWA to low‐amplitude fast (LAF) activity, similar to that seen in REM‐like sleep. The transition from SWA to LAF activity occurred simultaneously in all subregions of the mPFC, with no subregional‐dependent differences in the effects of electrical VTA stimulation. The local field potential transitions were associated with a change in the firing patterns of presumed pyramidal cells from phasic firing during SWA, with spikes restricted to the Up state, to a more continuous, tonic firing during the VTA stimulation‐evoked LAF activity. In both its spectral content, and in the associated firing pattern, the LAF activity evoked by VTA stimulation was very similar to the spontaneous LAF activity that occurs under urethane anaesthesia. The transition from SWA to LAF activity evoked by stimulation of the VTA was completely blocked by systemic administration of the dopamine D_1_‐like receptor antagonist SCH23390.

The majority (~60%) of VTA neurons which project to mPFC are dopaminergic, while the remainder are GABAergic and glutamatergic neurons (Gorelova, Mulholland, Chandler, & Seamans, 2012). Furthermore, some dopamine neurons also co‐release glutamate and GABA (Hnasko et al., 2010; Trudeau et al., 2014). Thus, the effect of the VTA stimulation‐evoked transition to LAF activity could potentially be mediated by dopamine, glutamate or GABA. A key finding of our study, however, was that the transition from SWA to REM‐like LAF activity, evoked by VTA stimulation, was blocked by the D_1_‐like receptor antagonist SCH23390. Thus, although our VTA stimulation did not activate a specific class of neurons, we can conclude that the observed effect on the local field potential in the mPFC was a consequence of dopamine release and dopamine receptor activation.

### 4.1 VTA firing patterns

In vivo, VTA neurons fire either with a slow (~1–5 Hz) tonic firing pattern, or with a phasic burst pattern with inter‐burst intervals up to 40–50 Hz (Grace, 1991). The frequencies of our VTA electrical stimulation conditions (25–50 Hz) were, therefore, selected to reflect the known VTA neuron activity patterns that have been recorded in vivo. We found that we could evoke the switch from SWA to LAF activity with stimuli of 25 or 50 Hz, and with regular or burst type patterns. Thus, we suggest that the effect of our VTA stimulation on the local field potential, and unit spiking activity in the mPFC, was evoked by physiologically relevant firing patterns of VTA neurons. Both the rate and firing pattern of VTA neurons impacts extracellular levels of dopamine in the forebrain, with higher levels of dopamine occurring during the burst firing pattern (Floresco, West, Ash, Moore, & Grace, 2003). We would, therefore, predict that all our VTA stimulation conditions should lead to significant levels of dopamine release in the mPFC.

### 4.2 Dopamine in the mPFC

The mPFC is heavily innervated by dopamine fibres, and D_1_‐like receptors are found in high density in the mPFC on pyramidal cells in layers 2/3, 5 and 6 (Radnikow & Feldmeyer, 2018). Our data demonstrated a dose‐dependent block of the transition to LAF activity following blockade of the D_1_‐like receptor with SCH23390, suggesting a clear role for D_1_‐like receptors in mediating the transition from SWA to LAF activity. We found that the transition to LAF activity resulted in a change in unit firing from spikes associated predominantly only with the Up state to a more persistent firing pattern. In addition, during both spontaneous LAF and VTA‐evoked LAF activity, firing patterns were significantly more regular as indicated by the decrease in CV. These data suggest VTA activation has a profound effect on the firing properties of mPFC pyramidal cells. Earlier intracellular recordings in the mPFC have demonstrated a role for the D_1_‐like receptors in modulating mPFC neuronal activity (Lewis & O'Donnell; Lavin & Grace, 2001; Onn & Wang, 2005). Lewis and O'Donnell (2000) reported that short trains of VTA stimulation elicited long‐lasting transitions to the Up state, which were reduced in duration by pretreatment with the D_1_‐like receptor antagonist SCH23390. Again, recording in the mPFC, Onn and Wang (2005) showed that brief (1 s) trains of high‐frequency VTA stimulation (10 – 50 Hz) evoked sustained membrane depolarisations (Up states) which were attenuated by SCH23390. Furthermore, catecholamine depletion blocked the prolongation of the VTA stimulation‐evoked Up states, again suggesting dopamine in the mPFC, acting via D_1_‐like receptors, plays a key role in the persistence of Up states (Onn & Wang, 2005).

Onn and Wang (2005) also showed that the depolarisation of mPFC neurons lasted a few seconds longer than the brief VTA stimulation. Thus, the slow return to SWA after VTA stimulation, that we observed in some cases, might reflect a similar prolonged depolarisation of mPFC neurons. Consistent with a prolonged effect of dopamine, Iwashita (2014) showed that brief, phasic high‐frequency (40–50 Hz) stimulation of the VTA in awake mice evoked a long‐lasting Ca^2+^ transient recorded in mPFC that persisted for 20–30 s. The long‐lasting Ca^2+^ transient was reduced approximately 50% by systemic application of the dopamine D_1_‐like antagonist SCH23390, but was not affected by the dopamine D_2_ antagonist eticlopride (Iwashita, 2014). Interestingly, this Ca^2+^ transient peaked 6 – 7 s after the onset of stimulation, suggesting there is a delay to peak effect. This is consistent with the delay to the transition to LAF activity we saw with some stimulation parameters. In summary, these studies are consistent with our findings that VTA stimulation abolished the slow oscillations and evoked a transition to LAF activity dependent on D_1_‐like receptors, despite electrical stimulation exciting both DA and non‐DA neurons in the VTA.

### 4.3 The role of the VTA and dopamine in sleep‐state transitions

During natural sleep, in both humans and rats, non‐REM sleep is characterised by SWA predominating in the local field potential or EEG, with fast (>15 Hz) network oscillations concentrated on the Up state and much less activity on the Down state. In contrast, during REM sleep, oscillations in the neocortex are more like the awake state with continuous activity in the theta (4–12 Hz), beta (15–30 Hz) and gamma (30–80 Hz) frequency bands. REM‐like sleep states, where activity also consists of predominantly low‐amplitude‐faster activity, can also be recorded under urethane anaesthesia (Clement et al., 2008; Fenik et al., 2012; Rukhadze et al., 2008; Sakata & Harris, 2012). Our findings suggest that electrical stimulation of the VTA induces a more activated state, with low‐amplitude‐faster oscillations, that are similar in spectral content to both the spontaneous alternations seen under urethane anaesthesia and natural REM sleep. Although, it is currently unclear whether the VTA‐evoked LAF, and the spontaneous transitions, share similar mechanisms (Clement et al., 2008; Rukhadze et al., 2008; Fenik et al., 2012; Sakata & Harris, 2012).

Interestingly, in studies in unanaesthetised rats, Dahan et al. (2007) showed that VTA neurons fired slowly during non‐REM sleep states but exhibited sustained (~30 s) burst firing patterns during REM sleep. More recently, recordings from dopamine‐containing VTA neurons showed larger Ca^2+^ transients during REM sleep than either awake or non‐REM sleep (Eban‐Rothschild et al., 2016). In addition to the increased burst firing of VTA neurons, dopamine concentrations in the mPFC have also been shown to be higher during REM sleep, than non‐REM sleep (Lena et al., 2005). Furthermore, electrical stimulation (30 s) of the VTA evoked theta oscillatory activity in the hippocampus, a characteristic of the REM‐like sleep state (Orzel‐Gryglewska, Kusmierczak, Majkutewicz, & Jurkowlaniec, 2012). Our data showing the delay to the onset of LAF activity after VTA stimulation is also consistent with the observation that VTA neuron burst firing started 10–20 s before the onset of the REM‐like sleep state (Dahan et al., 2007). It is, therefore, possible that the dopamine concentration must reach a threshold level before a transition to REM‐like activity can occur. These findings, taken together with the present data, suggest that VTA stimulation at specific frequencies releases a sufficient concentration of dopamine to switch neurons from non‐REM to REM‐like activity in the mPFC.

Although it has long been known that stimulants that increase dopamine levels are potent wake‐promoting drugs (Boutrel & Koob, 2004), the role of dopamine in sleep regulation remains controversial (Monti & Monti, 2007 for review). Intracerebroventricular infusion of a selective D_1_‐like agonist has been shown to increase the time awake (Isac & Berridge, 2003). With regard to SWA to REM‐like sleep‐state transitions, SCH23390 has previously been shown to increase slow wave sleep and decrease REM sleep (Monti, Fernandez, & Jantos, 1990), while mice in a hyperdopaminergic state, due to knock‐out of the dopamine transporter, exhibited an increase in REM sleep (Dzirasa et al., 2006). Furthermore, these authors found that dopamine depletion led to an increase in slow wave sleep, although these effects were mediated by D_2_ receptors not D_1_‐like receptors.

Several recent studies have led to a further reassessment of the role of dopamine in both sleep‐state transitions and sleep–wake transitions. Taylor et al. (2016) showed that optogenetic stimulation of VTA dopamineergic neurons was sufficient to restore consciousness to isoflurane anaesthetised mice and this arousal was blocked by systemic injection of the dopamine D_1_‐like antagonist SCH23390. Excitation of VTA neurons using chemogenetic control with DREADDs greatly increased the time mice spent awake (Oishi et al., 2017). However, in the Oishi et al. (2017) study, the increase in wakefulness was found to depend upon activation of D_2_ receptors as systemic injection of the D_2_ antagonist raclopride completely blocked the wake‐promoting effects, while the D_1_‐like antagonist SCH23390 only slightly attenuated the effect (although importantly SCH23390 was used at a lower concentration than this study). Thus, there is clear evidence that dopamine does play a role in sleep and arousal, although the relative contribution of D_1_‐like or D_2_ receptor subtypes varies between studies.

### 4.4 Non‐dopaminergic control of sleep‐state transitions

We cannot exclude the possibility that either glutamatergic or GABAergic neurons may also have played a role in the transition to LAF we have observed with VTA stimulation. Onn and Wang (2005) recorded long latency excitatory post‐synaptic potentials in PFC following VTA stimulation that could reflect glutamate co‐released from dopamine neurons in the VTA (Rayport, 2001; Chuhma et al., 2004). More recently, Yu et al. (2019) have shown that both glutamatergic and GABAergic neurons in the VTA promoted wakefulness which persisted after systemic injection of the D_1_‐like antagonist SCH23303. Overall, these data suggest that multiple neurotransmitter systems from the VTA may each play a role in sleep‐state or sleep–wake transitions. The situation is further complicated given that both direct and indirect pathways from the VTA could be activated.

### 4.5 Limitations of the current study

Although our data demonstrated a clear blockade of the VTA stimulation‐evoked transition to LAF activity following systemic application of the dopamine D_1_‐like receptor antagonist, we cannot rule out the possibility that complex multi‐synaptic connections contributed to this effect. VTA neurons project to multiple forebrain structures in addition to the mPFC (Ikemoto, 2007; Oades & Halliday, 1987). Intermediate structures such as the nucleus accumbens (NAc) or central nucleus of the amygdala (CeA), which project to the mPFC (Ikemoto, 2007; Oades & Halliday, 1987), could also play a role. Local infusion of the D_1_‐like antagonist into the PFC during VTA stimulation would confirm the role of D_1_‐like receptors in the mPFC in mediating the transition to LAF activity evoked by VTA stimulation. Furthermore, VTA stimulation could have antidromically activated brain regions that project both to, and through, the VTA and (directly or indirectly) to the mPFC. Whether the transition to LAF activity we observed is due to direct or indirect activation will be important to ascertain in the future as another recent study suggested that selective optogenetic stimulation of the VTA‐mPFC projection did not significantly alter non‐REM sleep duration (Eban‐Rothschild et al., 2016). The latter study proposed that the NAc projections played the most significant role in promoting wakefulness. However, it is possible that the pathways, neurotransmitters and receptor subtypes, involved in sleep‐state transitions may differ from those involved in sleep–wake transitions.

The VTA is also not the only midbrain region whose activation is able to evoke a transition to an LAF pattern of activity (reviewed in Jones, 2020). Stimulation of other brainstem structures, such as the pedunculopontine nucleus (Valencia, Artieda, Bolam, & Mena‐Segovia, 2013), cholinergic brainstem structures (Clement et al., 2008), and nucleus pontis oralis (Takataet al., 2018), have all been shown to evoke a sleep‐state transition to a more activated state. These data all indicate that multiple pathways, neurotransmitters and receptor subtypes, contribute to both the sleep‐state and sleep–wake transitions. Future studies targeting specific neuronal populations using optogenetics would help elucidate which neuronal population, and brain regions, were responsible for the transition to LAF evoked VTA by stimulation.

In addition, slow oscillations propagate and spread along the cortex as travelling waves in both humans (Massimini, Huber, Ferrarelli, Hill, & Tononi, 2004) and rodents (Ruiz‐Mejias et al., 2011). Therefore, it is highly likely that the VTA‐evoked transition to LAF could spread to cortical areas beyond the mPFC. However, future studies using multi‐site recordings across the cortex are needed to elucidate how the VTA stimulation‐evoked changes we observed in the mPFC affect other cortical areas, and whether dopamine D_1_‐like receptors also play a role in sleep‐state transitions in other cortical areas.

### 4.6 Future directions

The role of DA in sleep‐state and sleep–wake transitions has been re‐evaluated in recent years with several detailed studies, including those using optogenetic and DREADD chemogenetic approaches, demonstrating a role clear for dopamine. Many neuropsychiatric and neurodegenerative conditions are associated with changes in dopamine including schizophrenia (Grace, 2016) and Parkinson's disease (Michel, Hirsch, & Hunot, 2016). Both these conditions are also linked to abnormalities in the sleep–wake cycle (Amara, Chahine, & Videnovic, 2017; Monti et al., 2013). In fact, changes in the sleep–wake cycle may be central to the cognitive deficits associated with these conditions, especially in Parkinson's disease which is caused by a degenerative loss of dopamine neurons in the VTA and substantia nigra (see Michel et al., 2016 for review) and in which sleep changes occur decades before the onset of other clinical symptoms (Amara et al., 2017; Chahine, Amara, & Videnovic, 2017). A better understanding of the role of dopamine in sleep‐state and sleep–wake transitions will provide important insights into the sleep and memory disturbances associated with a wide range of neuropsychiatric and neurodegenerative diseases.

## CONFLICT OF INTEREST

The authors have no conflicts of interest, financial or otherwise, to declare.

## AUTHOR CONTRIBUTIONS

SG performed experiments; SG, SEG, BO and MS prepared analysis scripts and analysed data; SG, BO and SEG prepared figures; SG, AR, SEG, BO and FENL edited and revised manuscript; SG, AR, SEG and FENL approved final version of manuscript; AR, SEG, SG and FENL interpreted results of experiments; SEG and FENL drafted manuscript.

## Data Availability

All original data will be made available upon reasonable request.
